# Noninvasive Detection of Salt Stress in Cotton Seedlings by Combining Multicolor Fluorescence–Multispectral Reflectance Imaging with EfficientNet-OB2

**DOI:** 10.34133/plantphenomics.0125

**Published:** 2023-12-08

**Authors:** Jiayi Li, Haiyan Zeng, Chenxin Huang, Libin Wu, Jie Ma, Beibei Zhou, Dapeng Ye, Haiyong Weng

**Affiliations:** ^1^ College of Mechanical and Electrical Engineering, Fujian Agriculture and Forestry University, Fuzhou 350002, China.; ^2^ Fujian Key Laboratory of Agricultural Information Sensoring Technology, College of Mechanical and Electrical Engineering, Fujian Agriculture and Forestry University, Fuzhou, Fujian 350002, China.; ^3^ State Key Laboratory of Eco-hydraulics in Northwest Arid Region of China, Xi’an University of Technology, Xi’an 710048, Shaanxi, China.

## Abstract

Salt stress is considered one of the primary threats to cotton production. Although cotton is found to have reasonable salt tolerance, it is sensitive to salt stress during the seedling stage. This research aimed to propose an effective method for rapidly detecting salt stress of cotton seedlings using multicolor fluorescence–multispectral reflectance imaging coupled with deep learning. A prototyping platform that can obtain multicolor fluorescence and multispectral reflectance images synchronously was developed to get different characteristics of each cotton seedling. The experiments revealed that salt stress harmed cotton seedlings with an increase in malondialdehyde and a decrease in chlorophyll content, superoxide dismutase, and catalase after 17 days of salt stress. The Relief algorithm and principal component analysis were introduced to reduce data dimension with the first 9 principal component images (PC1 to PC9) accounting for 95.2% of the original variations. An optimized EfficientNet-B2 (EfficientNet-OB2), purposely used for a fixed resource budget, was established to detect salt stress by optimizing a proportional number of convolution kernels assigned to the first convolution according to the corresponding contributions of PC1 to PC9 images. EfficientNet-OB2 achieved an accuracy of 84.80%, 91.18%, and 95.10% for 5, 10, and 17 days of salt stress, respectively, which outperformed EfficientNet-B2 and EfficientNet-OB4 with higher training speed and fewer parameters. The results demonstrate the potential of combining multicolor fluorescence–multispectral reflectance imaging with the deep learning model EfficientNet-OB2 for salt stress detection of cotton at the seedling stage, which can be further deployed in mobile platforms for high-throughput screening in the field.

## Graphical abstract

**Figure:**
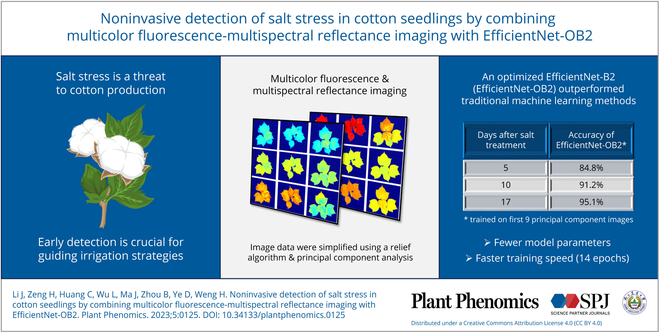

## Introduction

Cotton (*Gossypium hirsutum*) is considered as one of the most important industrial crops and widely cultivated in a diverse range of climatic conditions across the world [1], with a yield of 25,733,000 metric tons and an area of 33,187,000 ha in 2021 (www.icac.org). During cotton’s lifetime, it suffers from several biotic and abiotic stresses, among which salt stress has become one of the major threats for maintaining sustainable cotton yield. It was reported that as much as 60% of cotton yield was reduced as a result of salt stress [2]. There is a 10% increase in area of salt-effected land annually, and it was predicted that 50% of arable land will be salt effected by 2050 [3], which will gravely endanger the production of cotton.

Although cotton is regarded as a moderately salt-tolerant crop with a salinity threshold level of 7.7 dS m^−1^, it is prone to be more sensitive to salinity stress during the germination, emergence, and young seedling stages in comparison with later stages [4,5]. The adverse impact on morphological, physiological, and biochemical traits by salinity in the young seedling stage will ultimately induce a reduction of yield. As reported by Ma et al. [6], salinity can delay the emergence of cotton for up to 4 to 5 days on soil with an electrical conductivity of 15 to 20 dS m^−1^ compared with those normal ones. Additionally, the root, shoot growth, and shoot/root ratio can also be significantly inhibited by up 36.7% under high salt concentration in soil [7] as well as the number of bolls, boll weight, and fiber quality (length, strength, and micronaire values) [8]. Apart from morphological modifications, physiological response to salinity was observed with disruption in photosynthetic apparatus and activities and inorganic ionic balance. Jiang et al. [9] proved that salt stress can significantly limit the net photosynthetic rate, mesophyll conductance, and dark respiration rate of cotton leaf. Similar results can be found in Shen et al. [10] and Abuduwaili et al. [11] with a reduction of chlorophyll content and maximal quantum yield of PSII photochemistry (*Fv*/*Fm*). Excessive salt level caused ion toxicity and mineral perturbations, inhibited Ca absorption, and reduced the transport capacity of P, Mg, and Cu [12]. Non-invasive micro-test technology (NMT) also revealed that the net Na^+^ flux increased in mesophyll cells after NaCl solution irrigation [13]. Due to the adverse impact of salt stress on cotton growth, considerable efforts of management strategies of salt stress for cotton have been paid by manipulating salt-responsive genes, such as *GhMKK1* and *GhGRF* for developing salt-tolerant genotypes [14,15]. Additionally, magnetically treated brackish water was introduced to improve the soil microenvironment as well as enhance soil water retention and nutrient transformation with aim to alleviate salinity stress to cotton [16,17].

Early and rapid salt stress detection of cotton in salt-sensitive seedling stages plays a vital role in guiding irrigation strategies. Although destructive methods for obtaining physiological and biochemical molecular traits (malondialdehyde [MDA], superoxide [SOD], chlorophyll content, etc.) of cotton can accurately indicate the status of salt stress [18], they were labor intensive and inefficient. Fortunately, optical-based technologies have become mainstream approaches for sensing morphological, biochemical, and physiological traits of plants owing to their non-destructive, high-throughput, and objective nature [19,20]. The interaction between light and plant tissues involves reflection, transmission, and absorption, which can be sensed by optical sensors that closely depend on the biochemical and physiological characteristics within plants from different aspects under different growing conditions [21,22]. Consequently, electromagnetic responses at different wavelengths have been used to develop optical sensors such as red–green–blue (RGB), chlorophyll fluorescence (ChlF), and hyperspectral (HS), multispectral (MS), and thermal sensors for sensing different characteristics of plants’ health status. Spectral signatures from 400 to 1,000 nm were applied to diagnose salt stress in pomegranate at the asymptomatic stage with around 90% accuracy [23]. Drought tolerance evaluation in *Miscanthus sinensis* was carried out via multispectral imaging for fast and non-destructive quantifying of plant morphological and physiological responses under drought conditions [24]. Analysis of rapid chlorophyll fluorescence transient can serve as a non-destructive indicator of the level of salt stress in leaves for tomatoes with the changes in the values of JIP parameters and energy fluxes [25] and was also introduced for the detection of macronutrient deficiency in *Miscanthus* plants [26]. Tian et al. [27] and Yao et al. [28] found that multicolor fluorescence imaging served as a powerful tool to provide primary and secondary metabolic information in plants and can be used for non-invasive salt stress detection in Arabidopsis, as well as in *Jatropha curcas* L. [29]. Additionally, kinetic chlorophyll fluorescence imaging that can uncover the photochemical and non-photochemical components of photosystem II (PSII) was also applied to non-invasively detect drought and heat stress in strawberry and to understand the genetic architecture of water stress tolerance in *Lactuca sativa* L. [30]. The above-mentioned studies demonstrated that optical-based sensing methodologies can enable a high-throughput analysis to interpret how optical-based traits related to plant biochemical and physiological activities respond to biotic and abiotic stresses. However, it is costly and multi-source images relating to different characteristics of the plant excited by different light sources cannot be obtained synchronously under the field of view of one optical sensor. Additionally, to the best of our knowledge, few researches have been carried out to explore the potential of optical-based sensing for noninvasively detecting salt stress of cotton seedlings.

The rapid and noninvasive detection of salt stress is critical from the standpoint of cotton management at the seedling stage as it allows high-throughput screening of cotton status. This goal is pursued through the following steps: (a) Considerable efforts have been made to develop an automated, high-throughput system to obtain multicolor fluorescence and multispectral reflectance images synchronously under the field of view of one optical sensor; (b) the evaluation of the physical, chemical, and photosynthetic traits corresponding to responses to salt stress at the seedling stage is being conducted; and (c) the feasibility of early salt stress detection in cotton seedlings through deep learning is under assessment. Overall, this work is expected to provide a guideline for cotton irrigation management strategies, with the potential to enhance yields, while also presenting potential solutions for other applications in plant phenotyping.

## Materials and Methods

### Cotton seedlings and growth conditions

**Fig. 1.**
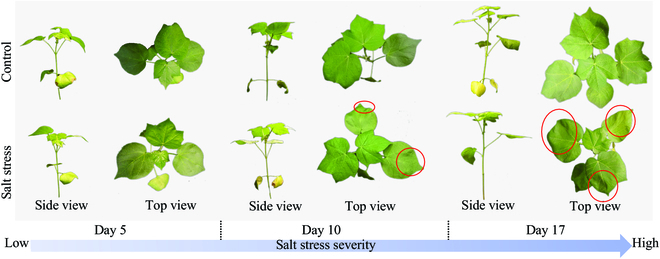
Images of control and salt-stressed cotton seedlings at days 5, 10, and 17, respectively.

The genotype of *cv. Xinluzao*58 was selected for screening salt stress, which was cultivated in the greenhouse at Fujian Agriculture and Forestry University in Fuzhou, Fujian province. Seeds were sown in plastic pots (90 mm in diameter) containing quartz sand with a particle size greater than 0.25 mm to ensure air permeability for plant roots. Seeds were germinated at 40% to 60% humidity with 300 μmol·m^−2^·s^−1^ PPF (photosynthetic photon flux) under full-spectrum light-emitting diodes (LEDs; Full spectrum–120 cm–80 W, Shandong Guixiang Optoelectronics Co., Ltd) fixed 1 m away from pots. The day/night temperature was 35/28 ^°^C with a photoperiod of 14/10 h. Once the seedlings emerged, 100 ml of nutrient solution (Hoagland modified nutrient solution) was applied into each pot from 9 to 10 PM every night. At the beginning of the 3-leaf stage, the cotton seedlings were divided into 2 groups with 101 and 102 pots for salt treatment and control, respectively. To avoid deadly salt shock, the salt treatment on cotton seedlings was initiated with 200 ml of nutrient solution with an increasing NaCl concentration of 25 mM every day until the fourth day when NaCl concentration reached 100 mM. The control group followed the same methodology but without the application of NaCl. The visible images of cotton seedlings at different experimental dates are shown in Fig. 1. No obvious difference between control and salt-stressed cotton seedlings can be found by the human eye after the application of NaCl solution for 5 days while some curling leaves appeared on cotton seedlings after 10 and 17 days’ treatment.

### Measurement of biochemical characteristics

Seventeen days after the salt stress treatment, typical biochemical characteristics including total chlorophyll, MDA, SOD, and catalase (CAT) were investigated, which allowed evaluating the response of cotton seedlings to salt stress. Thirty mature unfolded leaves of each group were randomly collected and frozen using liquid nitrogen immediately after detachment, for chlorophyll, SOD, and CAT measurements, respectively. The total chlorophyll content of 0.5 g of cotton leaf tissue was extracted using 50 ml of ethanol, and the absorption at 470, 649, and 665 nm of solution was determined using a microplate reader (M200 PRO, Tecan, Austria). The MDA, CAT, and SOD values of the sample were measured using the MDA kit (Solarbio, China), CAT kit (Solarbio, China), and SOD kit (WST-8 method, Grace Biotechnology, China), respectively. In the determination of MDA, the MDA in the sample extraction solution was subjected to condensation with thiobarbituric acid under 100 ^°^C acidic conditions. This reaction generated a brownish-red oxazolidinedione with a maximum absorption wavelength at 532 nm. By performing colorimetric analysis, the MDA content in the sample can be estimated. The measurement of CAT involved exploiting the ability of CAT to decompose hydrogen peroxide (H_2_O_2_) and the characteristic absorption peak of H_2_O_2_ at 240 nm. The CAT activity can be calculated based on the rate of change in absorbance. In the measurement of superoxide dismutase (SOD), WST-8 was employed to react with the superoxide anion (O^2−^) catalyzed by xanthine oxidase with the production of a water-soluble formazan dye. Since SOD catalyzed the disproportionation of the superoxide anion, this reaction step was inhibited by SOD. Therefore, the activity of SOD was inversely related to the amount of formazan dye, which can be determined through colorimetric analysis of the WST-8 product. During the measurement of each biochemical indicator, 3 technical replicates were carried out and the mean value was used for later analysis.

### Phenotyping platform development

**Fig. 2.**
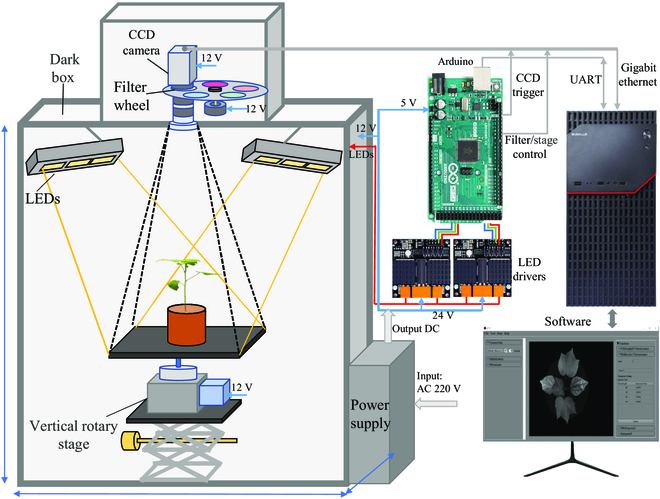
Overview of the developed phenotyping platform.

Advanced optical sensors not only can non-destructively monitor the physical properties of plants (i.e., growth and structure) but also, to a great extent, can gain insight into their functional, molecular, and biophysical dynamics responding to the interaction between genotypes with environmental factors [31,32]. Different architectures of phenotyping platforms have been developed with specific goals [32–34]. However, most phenotyping platforms can only quantify the reflectance, absorption, or transmittance of light within leaves or canopy independently from different sensors, which makes it difficult to obtain this information synchronously as well as to fuse data with different spatial/spectral resolutions. To address the need for synchronously obtaining reflectance and fluorescence images of cotton seedlings with the same resolution to gain more comprehensive physical and biological traits for interpreting the impact of salt stress, effort has been made by developing an affordable image-based phenotyping platform. The reflectance was regarded closely as a chemical characteristic of plants while multicolor fluorescence images related to the primary and secondary metabolites and photosynthetic activities [35,36]. The prototyping platform was modified from our previous version that can take images of each cotton using the “plant-to-sensor” method [27]. The overview of the developed phenotyping platform is shown in Fig. 2.

**Fig. 3.**
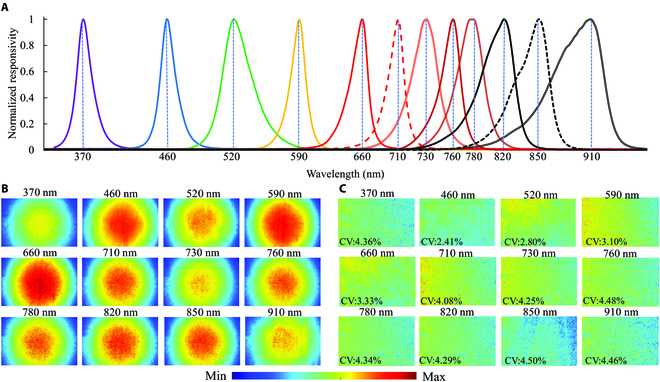
Optical spectra of the 12 LEDs used in this platform (A). Light distribution of each wavelength before (B) and after (C) correction. CV represents the coefficient of variation.

In general, the LED panel includes 12 narrow-band LEDs at central wavelengths of 370, 460, 520, 590, 660,710, 730,760, 780, 820, 850, and 910 nm, respectively (Fig. 3A). Among them, ultraviolet (UV) LEDs (370 nm) consist of 8 panels with a power of 50 W for each array in the upper right corner of the cotton seedlings to provide uniform and enough UV radiation, which can excite a blue and green fluorescence of cotton leaf tissue as well as a red and far-red chlorophyll fluorescence with maxima wavelength near 440, 520, 690, and 740 nm, respectively [37]. The other 44 LEDs with 4 LEDs for each wavelength were introduced to provide the power of 12 W to illuminate cotton for collecting reflectance images at corresponding wavelengths that were triggered in sequence according to commands from a microprogrammed control unit (Arduino Mega 2560, Arduino, Italy). The light distribution of each wavelength is shown in Fig. 3B. The coefficient variation of light distribution within the corrected image of each wavelength (Fig. 3C) was in the range from 2.41% to 4.50%, indicating that light distributed uniformly on this platform. A monochrome CMOS (Complementary Metal Oxide Semiconductor) camera (MV-CA020-20GM, Hikvision, Hangzhou, China) with high quantum efficiency (QE) in the near-infrared range was used to collect those images (1,920 × 1,200 pixels) with a high signal-to-noise ratio (SNR). A filter wheel with 5 narrow-band filters was installed between the lens (M0812, Computar, Japan) and the CMOS camera. Among them, 4 narrow-band filters (D10-M82, CONTAX, China) with a full width at half maximum of 30 nm at maxima wavelength near 440, 520, 690, and 740 nm allowed blue (F440), green (F520), red (F690), and far-red (F740) fluorescence to enter the CMOS camera, respectively, excited by UV radiation. The left one was a long-pass (400 to 1,000 nm) filter to ensure the same focal distance for multispectral reflectance imaging as multicolor imaging. The rotation of filter on the filter wheel was controlled by the step motor (ZD-M42P, Hongbaoli Electronic, Shenzhen, China). A Windows-based software was developed in C++ in conjunction with the software development kit provided by the CCD manufacturer for image collection by sending/starting the command to the Arduino via UART communication after setting optimal parameters (gain, exposure times of each image, and filename).

### Images acquisition by the prototyping platform

The images of each pot were captured between 10 AM and 3 PM to ensure a consistent inclination of cotton leaf after full light adaption in an artificial climate greenhouse. Prior to image acquisition, the cotton was put on an adjustable vertical rotary stage with a working distance of 45 cm from the lens to the canopy. The multicolor fluorescence imaging of cotton seedlings was carried out firstly followed by multispectral imaging. The exposure times for F440, F520, F690, and F740 were 350 ms, 250 ms, 100 ms, and 100 ms, respectively, with a gain of 6. For multispectral imaging, the exposure times for R460, R520, R590, R660, R710, R730, R760, R780, R820, R850, and R910 were 500, 100, 100, 50, 80, 80, 40, 40, 50, 50, and 50 ms, respectively, with a gain of 8, to gain the reflectance image at each waveband with the best SNR due to different QE during 400 to 1,000 nm. During the data acquisition, the sampling sequence of the control and the salt stress groups was crossed over at intervals of 3 pots to avoid significant interference errors with the time going. To avoid the impact of leaf inclination angle on the image pattern, 4 top-view images of each cotton seedling were collected for analysis on a stage in the platform by rotating every 90°. The mean spectral reflectance at each wavelength of each cotton seedling was calculated by the averaging values at 4 positions. The reference images of the plate covered with gray latex paint produced by Nippon company were acquired every 2 h during this process for cotton seedling image correction caused by uneven light distribution using Eq. 1:

$$R_{j}=\frac{S_{j}-D_{j}}{W_{j}-D_{j}}\times 100\%$$ (1)

where *R_j_*, *S_j_*, *D_j_*, and *W_j_* represent corrected cotton image, original cotton image, dark current, and reference image, respectively, at *j*band. The whole cotton was considered as the region of interest after removal of the background by applying a mask generated from F740.

### Data analysis

#### Vegetation index calculation

Changes in biochemical content and cellular structure of salt stress on cotton leaves are responsible for the corresponding spectral reflectance patterns. To further utilize these spectral features, 3 vegetation indices with potential sensitivity to the salt-induced changes were selected because they were proved useful for plant health status inspection, such as greenness (GR) [24], plant senescence reflectance index (PSRI) [38], and normalized difference vegetation index (NDVI) [39]. They were described as follows:

$$\mathrm{GR}=\left(2^{*}R_{\text{Green}}-R_{\mathrm{Red}}-R_{\text{Blue}}\right)/\left(2^{*}R_{\mathrm{Red}}+R_{\text{Green}}+R_{\text{Blue}}\right)$$ (2)

$$\text{PSRI}=\left(R_{\mathrm{Red}}-R_{\text{Green}}\right)/R_{\mathrm{NIR}}$$ (3)

$$\text{NDVI}=\left(R_{\mathrm{NIR}}-R_{\mathrm{Red}}\right)/\left(R_{\mathrm{NIR}}+R_{\mathrm{Red}}\right)$$ (4)

where R_Blue_, R_Green_, R_Red_, and R_NIR_ were the reflectance at wavelengths of 450, 520, 660, and 780 nm, respectively.

#### Reduction of data dimension using principal component analysis

The prototyping platform was able to collect reflectance and fluorescence images of cotton synchronously, which could obtain 79 parameters after the mathematical operation, including basic parameters, simple ratio (SR) indices, and vegetation indices, which might contain redundant features in feature space. In this study, the Relief algorithm was then introduced to find a feature subset that was most relevant to the salt detecting model by estimating feature weights according to their ability to discriminate salt-stressed from control cottons [40]. It has been successfully applied to reduce model complexity during plant biotic and abiotic detection [41,42]. After important features selected by the Relief algorithm, principal component analysis (PCA) was further implemented on one-dimensional original variables to maximize the representation of salt-stressed and control cottons by observing few principal components (PCs) in a new feature space through orthogonal transformation without largely losing useful information [43]. For a 3D image cube (combination of multispectral and multicolor fluorescence images), PCA can also be used to reduce spectral dimensions by unfolding each single-band image into a vector and thus the 3D image cube was reshaped to form a 2D matrix that PCA can successfully process. After PCA weight average, each score vector of selected principal components was folded back into 2D images that would enhance the contrast of features between salt-stressed and control cottons. The first several principal components that explained 95% of the original variance were later used for analysis.

#### Model construction for the detection of salt stress of cotton seedlings

In this study, 3 commonly used models, including K-nearest neighbor (KNN), support vector machine (SVM), and random forest (RF), were built based on selected one-dimensional features to investigate their ability for salt stress detection of cotton seedlings. Generally, the KNN algorithm first finds *k* nearest neighbors of a query during the training process, and then predicts the query based on the majority vote of its *k* nearest neighbor [44]. SVM works by finding a hyperplane where it can separate the 2 classes while maximizing the distance between the hyperplane and boundary training samples, which can finally solve a convex quadratic programming problem [45]. RF is an ensemble supervised learning technique that constructs a series of tree-structure classifiers. Every tree is planted according to a training sample set with a random variable and each tree casts a unit vote for the most popular class. The final result was based on their combined results [46]. These 3 machine learning models have been widely used for the identification of plant stress and disease detection [47,48]. In contrast to those 3 classical approaches, deep learning was also introduced in this task because it can actively learn a variety of parameters from raw images without any hand-tuned pre-processing steps during the model training process [49]. This study employed the general EfficientNet-B0 network designed by Tan and Le through neural architecture search to design a new baseline network including layers, channels, node connections, activation function, and so on [50], which presented a robust performance across multiple datasets, such as ImageNet, CIFAR-100, and Flowers. The EfficientNet-B0 network was considered as a deep learning model that can be can be deployed on commercial edge devices with a fixed resource budget. The compound scaling method was utilized to determine the optimal depth, width, and input resolution of the network while working under a fixed resource constraint. Equations 5 to 8 provided a compound scaling method as follows:

$$\text{depth}:d=\alpha^{\phi }$$ (5)

$$\text{width}:w=\beta^{\phi }$$ (6)

$$\text{resolution}:r=\gamma^{\phi }$$ (7)

$$s.t.\alpha ∙\beta^{2}∙\gamma^{2}\approx 2\;\left(\alpha \geq 1,\beta \geq 1,\gamma \geq 1\right)$$ (8)

where *ϕ* is a specified value of 1 for EfficientNet-B0 due to twice more resources available, while *α*, *β*, and *γ* represent constants that determine how to distribute these extra resources to network width, depth, and resolution, respectively. According to the authors [50], they used neural architecture search as the compound scaling method to obtain the optimal values of *α*, *β*, and *γ* for EfficientNet-B0 with 1.2, 1.1, and 1.15, respectively, which can satisfy the constraint of *α·β^2^·γ^2^*≈2. Under the given resource constraints, the EfficientNet-B0 maximized the discriminant accuracy formulated as an optimization problem as Eqs. 9 to 12:

$$\;\begin{array}{c} \max\\ d,w,r \end{array}\;\text{Accuracy}\left(\mathrm{Net}\left(d,w,r\right)\right)$$ (9)

$$s.t.\mathrm{Net}\left(d,w,r\right)={⊙}_{i=1\ldots s}{\hat{F_{i\;}}}^{d∙\hat{L_{i\;}}}\left(X_{<r\cdot \hat{H_{i}},\hat{r∙W_{i},}w\cdot \hat{C_{i}}>}\right)$$ (10)

$$\text{Memory}\left(\mathrm{Net}\right)\leq \text{terget}\_\text{memory}$$ (11)

$$\text{FLOPS}\left(\mathrm{Net}\right)\leq \text{terget}\_\text{flops}$$ (12)

EfficientNet-B0 used Swish as an activation function instead of the rectifier linear unit (ReLU). The mini batch size, learning rate, and optimizer were 128, 0.01, and Adam, respectively. The performance evaluation of the models is based on accuracy, recall, precision, and F1 score. Recall (Sensitivity) measures the proportion of actual positive instances that are correctly identified as such by the model, and precision reflects the ability of the model to identify only relevant instances among all instances it labeled as belonging to the positive class. The F1 score considers the harmonic mean of precision and recall, balancing both factors. It is calculated as Eq. 13:

$$F1=2∙\frac{\text{Precision}∙\text{Recall}}{\text{Precision}+\text{Recall}}$$ (13)

**Fig. 4.**
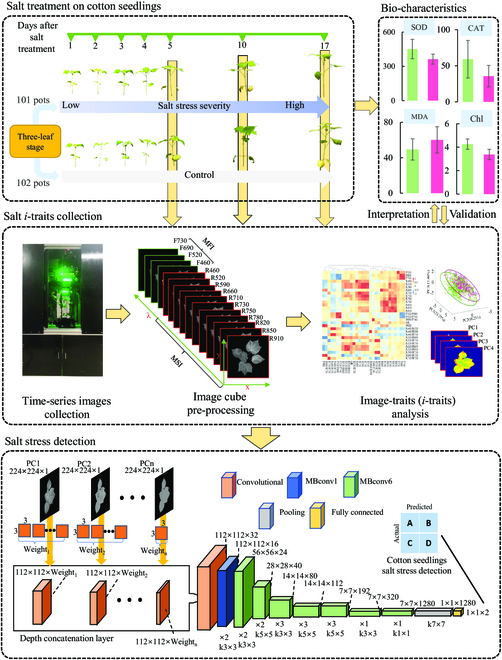
Schematic overview of the analytical procedure for cotton seedlings’ salt stress detection.

The total number of parameters that can be learned for baseline EfficientNet-B0 is 4,031,118 when the input channel of image of each sample was 3. Since the number of input channels only affects the number of first convolutional kernels in the CNN structure, an increasing number of channels of the image of each sample would cause an increase in learnable parameters of *n*×3×3×32 (*n* is the number of increased channel). The detailed description of the thorough analytical flowchart is summarized in Fig. 4. Finally, a total of 2,436 samples (203×4 positions×3 periods) after 5, 10, and 17 days’ salt stress were obtained to develop the classification model. For each experimental period, 152 pots were randomly selected for model training and the remaining 51 pots were selected for model validation. Furthermore, data augmentation was also applied by flipping the images of training sets on the *X* and *Y* axes, stretching 75% to 125% of the images, and shifting within the imaging boundary for CNN training. Overall, there were 1,824 samples (152×4 positions×3 periods) and augmented data for training sets and 612 samples (51×4 positions×3 periods) for validation sets in the dataset. All the data analysis and model construction were implemented in MATLAB 2022b (The MathWorks, Inc., Natick, MA, USA).

## Results and Discussion

### Salt stress on biological characteristics of cotton seedlings

**Fig. 5.**
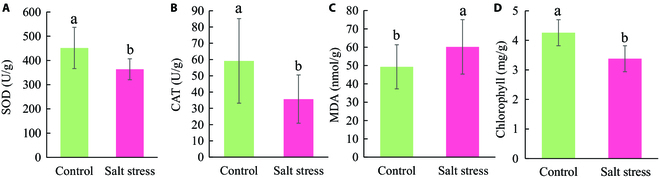
Effect of salt stress on the concentrations of superoxide (SOD) (A), catalase (CAT) (B), malondialdehyde (MDA) (C), and total chlorophyll content (D) in cotton seedling leaves after 17 days’ treatment. Data were shown as mean ± standard error (*n* = 10). Different letters indicated significant difference at *P* < 0.05 based on the Duncan test. The same below (Figs. 6 and 7).

Physiological changes in cotton seedlings due to the ionic toxicity of salt may assist us in understanding the response of cotton under saline conditions. The biological responses to salinity of cotton were expressed using SOD, CAT, MDA, and chlorophyll content in this study as shown in Fig. 5. After the 17 days of salt treatment, the activities of SOD and CAT decreased by 19.5% and 39.6%, respectively, as well as 26% for total chlorophyll content. However, MDA content presented an opposite pattern that increased by 22%. SOD and CAT are the key protective enzymes for the plant protecting against oxidative stress under adverse conditions, with an increasing pattern in early NaCl stress but a decreasing pattern in response to the stress extension [51,52]. In this study, the decreasing activities of SOD and CAT were caused by the scavenging ability of oxidative stress for cotton that was inhibited in terms of 17-day 100 mM salt stress [53,54]. An accumulation of MDA in cotton leaves was induced by the oxidative damage to the cell membrane. As chlorophyll content plays a key indicator to evaluate the photosynthesis capacity of the plant, a reduction in chlorophyll content in salt-stressed cotton might be due to the absorption of excessive amount of Na^+^ and Cl^−^ that leads ion imbalance, which can degrade chlorophyll content [22]. The changes in biological characteristics within cotton allowed a non-destructive detection of salt stress via multicolor fluorescence and multispectral imaging, which can reveal the structure and physiological status of a plant.

### Alterations in multicolor fluorescence of cotton seedlings upon salt stress

**Fig. 6.**
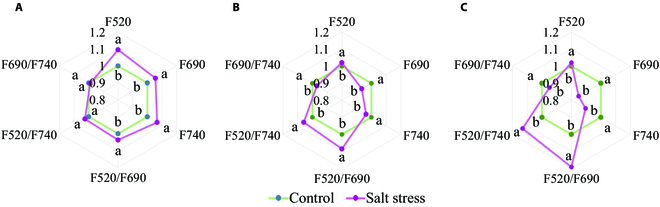
Changes of basic multicolor fluorescence parameters and their ratios after 5 (A), 10 (B), and 17 (C) days of salt stress treatment.

Multicolor fluorescence imaging was considered as a valuable probe of secondary metabolites and photosynthetic activities of a plant by inducting fluorescence emission using UV excitation [35]. In this research, the blue fluorescence (F440) emitted by cotton leaf was not taken into analysis due to a bad SNR. Therefore, the other 3 basic multicolor fluorescence with a central wavelength at 520 nm (F520), 690 nm (F690), and 740 nm (F740) were used for analysis. The difference of F520 between salt stress and control cotton seedlings was significant at day 5 (*P* < 0.05) but was not obvious at days 10 and 17 (*P* > 0.05), which demonstrated that it may play an important role in plant defense from stress at an early stage. It was found that the intensity of F690 and F740 increased with the extension of stress and presented a significant difference (*P* < 0.05) between control and salt-stressed cotton seedlings. The intensities of F690 and F740 of cotton seedling leaves in salt stress treatment were higher than control treatment after 5 days’ salt treatment, which generally might be caused by a short-term salt stress shock [55]. However, F690 and F740 of salt-stressed cotton were significantly decreased after 10 days’ salt treatment and decreased to a larger extent after 17 days compared with control ones. It indicated that the synthesis of chlorophyll was inhibited and began to decompose during salt stress. In fact, due to the spatial heterogeneity of fluorescence yield on the individual fluorescence image over the leaf area, fluorescence ratio images were often more reliable with fewer gradients. As shown in Fig. 6A, it generally caused an obvious increase in F520/F690 and presented a significant difference between stressed and controlled cottons after 5 days of salt stress treatment, which was not observed in F520/F740 or F690/F740. The difference in F520/F690 became more obvious after 10 and 17 days’ treatment, which was 1.085 and 1.192 times higher than control ones (Fig. 6B and C). It indicated that F520/F690 might be a more valuable and sensitive proxy for detecting salt stress in the early stage. During salt stress exposure, the fluorescence ratios of F520/F740 also increased while F690/F740 decreased in comparison with control ones. Therefore, the results demonstrated that salt stress resulted in the photosynthesis and secondary metabolites of cotton seedlings.

### Multispectral reflectance and vegetation indices analysis

**Fig. 7.**
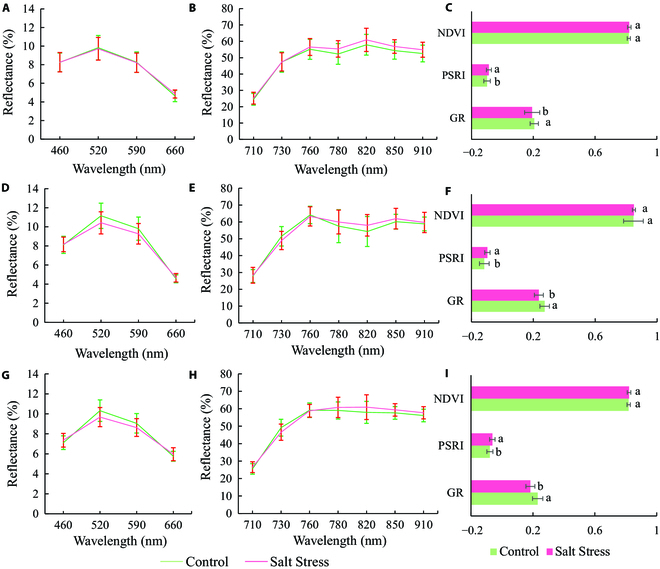
Mean reflectance and vegetation indices of control and salt-stressed cotton seedlings after 5 (A to C), 10 (D to F), and 17 (G to I) days of salt stress treatment.

The spectral reflectance of leaf can effectively provide the physiological and biological information about plants [36]. The mean reflectance of salt stress and control cotton seedlings at different treatment days is shown in Fig. 7. In the visible region, the reflectance at 520 nm and 590 nm of stressed samples significantly decreased after 10 days of salt stress treatment. In the near-infrared region, the reflectance of salt-stressed leaves from 780 nm to 910 nm was higher than that of control over time, which was consistent with previous studies that utilized a commercial hyperspectral imaging system [56]. Three vegetation indices are commonly used to monitor salt stress in cotton seedlings as shown in Fig. 7C, F, and I. Among these 3 vegetation indices, NDVI did not show a significant difference between the salt-stressed and control groups over time, while GR and PSRI were significantly different with a decrease in GR index but an increase in PSRI caused by salt stress. Similar results of a better sensitivity of PSRI, relating to the chlorophyll and carotenoid ratio, to abiotic stress than that of NDVI can also be found in a previous study that tracked leaf and fruit senescence [57]. The changes in pattern of GR and PSRI indicated that GR and PSRI were valuable proxies for the diagnosis of salt stress in cotton seedlings.

Basic reflectance images at corresponding wavebands collected by our phenotyping system were further used for calculation of 55 SR indices. The importance of each index was evaluated by the Relief algorithm. The important indices usually showed a high absolute weight value as shown in Table 1. According to the key idea behind Relief [40], it estimated feature relevance depending on how well feature values distinguish CK from salt-stressed cotton seedlings. In practice, the number of selected features should flexibly adapt to functional or computational conditions of the downstream modeling algorithms by removing as many of the irrelevant features as possible. In this study, the parameters with the top 5 highest weights after 5, 10 and 17 days of salt stress treatment, respectively, were selected according to a clear-cut separation between R460/R730 and R460/R590 at day 17. Therefore, a total of 11 SR parameters, including R460/R520, R460/R590, R460/R730, R520/R660, R520/R710, R520/R730, R520/R850, R520/R910, R590/R710, R660/R730, and R710/R730, were finally used for further analysis.

**Table 1.** The weight values of the top 10 simple ratio (SR) indices evaluated by the Relief algorithm

Day 5		Day 10		Day 17	
Parameters	Weights	Parameters	Weights	Parameters	Weights
R520/R730	0.009377	R520/R660	0.021317	R460/R520	0.047395
R520/R850	0.006789	R460/R590	0.019582	R520/R710	0.04658
R590/R710	0.006761	R460/R520	0.019379	R590/R710	0.044099
R660/R730	0.006675	R520/R710	0.018775	R710/R730	0.042844
R520/R910	0.006542	R660/R730	0.018075	R460/R730	0.041924
R520/R660	0.006372	R520/R590	0.016023	R460/R590	0.028346
R520/R780	0.006123	R590/R710	0.015362	R520/R910	0.026957
R520/R820	0.00609	R660/R760	0.014584	R710/R780	0.026881
R460/R710	0.006087	R520/R910	0.012751	R520/R850	0.025527
R520/R760	0.006038	R460/R730	0.012533	R710/R760	0.024179

### PCA on multicolor fluorescence and multispectral reflectance data

**Fig. 8.**
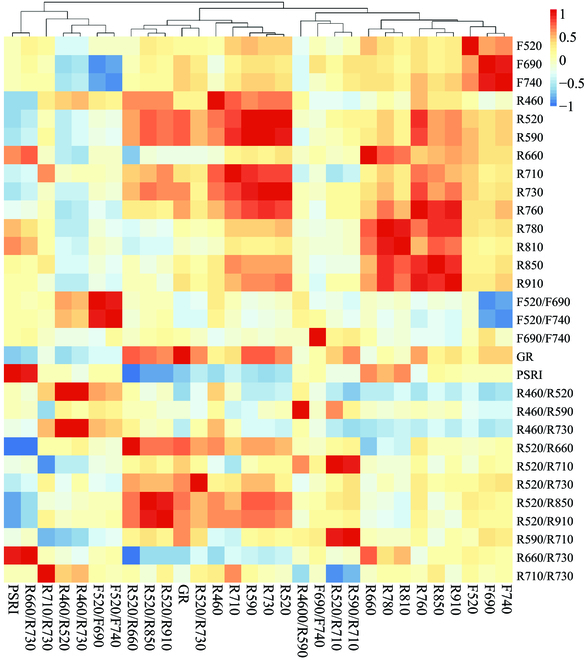
Heat map of Pearson’s correlation coefficients among different parameters.

Before PCA, Pearson correlation analysis was carried out on multicolor fluorescence parameters and multispectral reflectance parameters to further understand the correlation among various parameters. Figure 8 shows that the Pearson correlation coefficient among different multispectral reflectance parameters was from −0.96 to 0.97, and it was from −0.85 to 0.93 for multicolor fluorescence parameters. It illustrated a strong correlation internally for both multispectral reflectance parameters and multispectral reflectance parameters, indicating a need for dimensionality reduction. However, the Pearson correlation coefficient between fluorescence parameters and reflectance parameters was not as high as their internal ones, ranging from −0.61 to 0.51, indicating that the combination of these 2 types of data would enrich data for cotton seedlings’ salt stress detection.

**Fig. 9.**
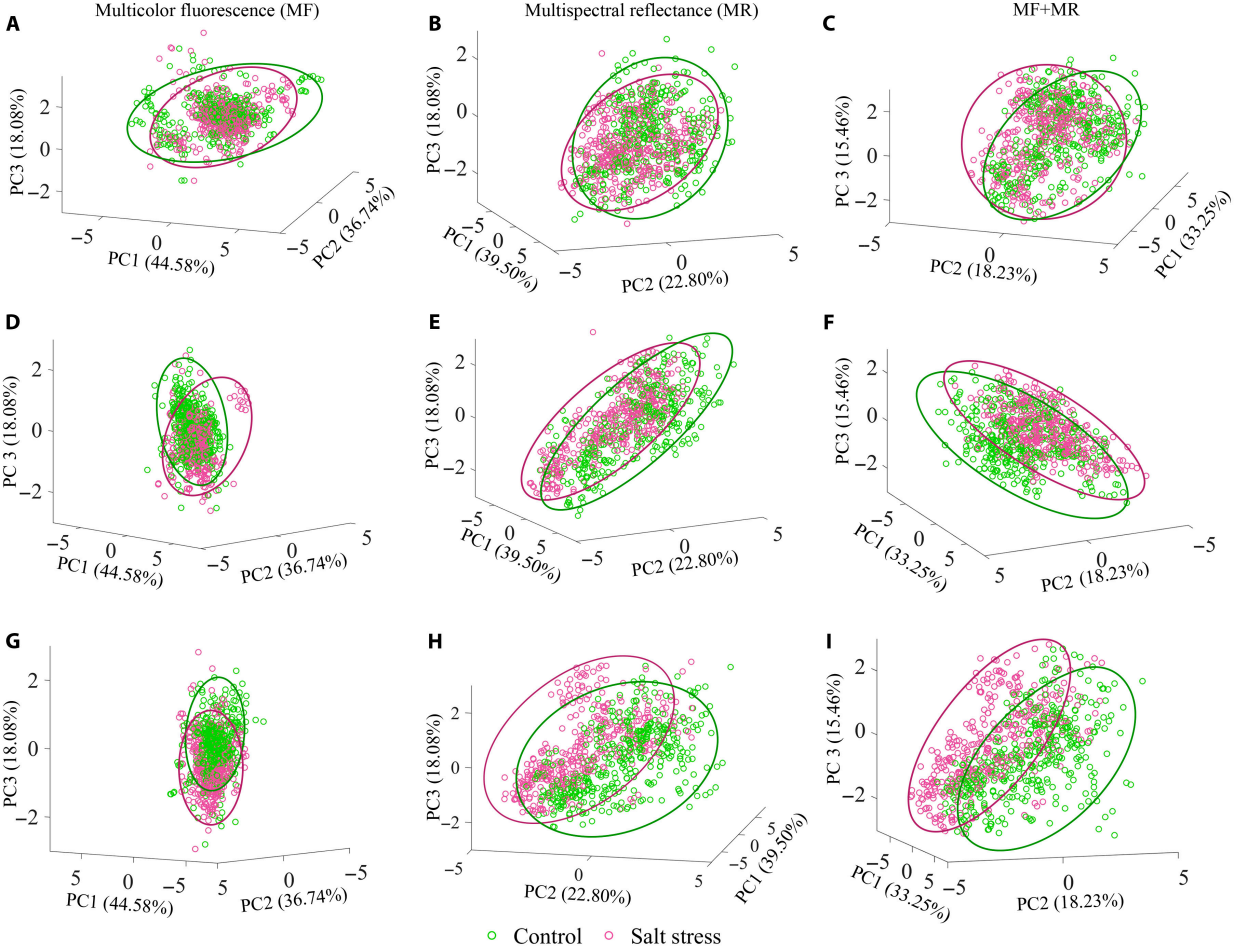
The overview of the systematic variation between control and salt-stressed cotton seedlings according to principal component 1 (PC1), 2 (PC2), and 3 (PC3) using multicolor fluorescence, multispectral reflectance parameters, and their combination, respectively, after 5 (A to C), 10 (D to F), and 17 (G to I) days of salt stress treatment.

The parameters including multicolor fluorescence data and multispectral reflectance data normalized together were subjected to PCA to obtain a preliminary overview of the systematic variation between control and salt-stressed cotton seedlings at days 5, 10, and 17, respectively. Based on visual inspection, the first 3 principal components extracted from multicolor fluorescence parameters, multispectral reflectance parameters, and their combination could explain 99.2%, 80.8%, and 66.9%, respectively, of the original variance after 5 (Fig. 9A to C), 10 (Fig. 9D to F), and 17 (Fig. 9G to I) days of salt stress treatment, respectively. In particular, the principal components playing an important role in separating control from salt-stressed cottons could be determined according to the separation trend. The separation trend becomes more obvious with the extension of salt stress but with some overlaps. Moreover, it can be observed that the separation trend was much better when PCA was performed on fluorescence data combined with multispectral reflectance data than individually. It also needs to be mentioned that the separation trend being not satisfying enough might be caused by the ineffectiveness of the unsupervised PCA method. Therefore, supervised approaches still needed to be employed to further accurately detect salt stress in cotton seedlings.

**Fig. 10.**
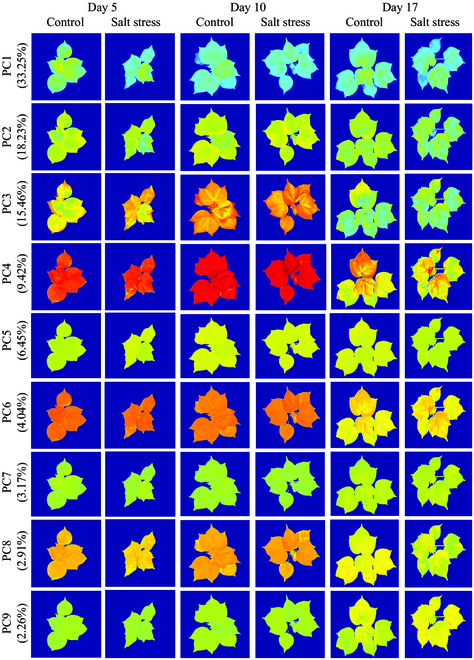
The first 9 principal component images were obtained from principal component analysis from the combination of multicolor fluorescence and multispectral reflectance images.

PCA was performed not only on the mean value of all samples (Table S1) but also on images that contained 30 channels for each sample by combining 3 original multicolor fluorescence images, 3 ratio fluorescence images, 11 multispectral reflectance images, 2 vegetation index images, and 11 SR images with the aim of obtaining spectral and spatial information synchronously under salt stress (Fig. 10). A quick visual inspection of principal component images revealed that the first 9 principal components can describe 95.2% of raw images without significant loss but can reduce 70% variables ((21/30)×100%). Compared to different principal component images, the first 4 principal component images provided relatively more texture details about leaves, while the others were more uniform in the distribution of pixels’ intensities. Overall, to optimize the model input, the different score images were tested to train discriminant models.

### Model establishment of salt stress detection for cotton seedlings

The detecting performance of cotton seedlings’ salt stress using SVM, RF, and KNN using data from multicolor fluorescence, multispectral reflectance data, and their combination, respectively, as shown in Table 2. Model establishment on multispectral reflectance generally achieved better results than multicolor fluorescence data. By combining multicolor fluorescence with multispectral reflectance data, the overall detecting accuracies of these 3 models had all significantly improved with a maximum of 28.24%, 20.10%, and 21.56% for 5, 10, and 17 days of salt stress detection, respectively, which indicated a necessity for combining multicolor fluorescence imaging and multispectral reflectance imaging for cotton seedlings’ salt stress detection. Among the different models, KNN presented the best ability for salt stress detection with overall detecting accuracies of 80.88%, 83.33%, and 85.78% for cotton seedlings after being subjected to 5, 10, and 17 days of salt stress, respectively. Effort was also carried out by building a model on the first 9 principal components from PCA on multicolor fluorescence data combined with multispectral reflectance data It can be found that the discriminant performance of PCA-RF and PCA-SVM slightly decreased. In contrast, the PCA-KNN models remained much robust with 2.08% improvement for the whole experimental period (5 to 17).

**Table 2.** The detecting performance of SVM, RF, and KNN trained from different data for salt stress of cotton seedlings after 5, 10 and 17 days’ salt treatment. The parameters used for the models are listed in Table S2.

Data	Day	SVM (%)	RF (%)	KNN (%)
Full multicolor fluorescence (MF)	5	50.49	56.37	52.45
	10	60.29	69.12	71.57
	17	69.12	65.69	64.22
	5–17	66.01	68.63	62.09
Full multispectral reflectance (MR)	5	74.02	78.92	72.06
	10	78.43	79.90	76.47
	17	81.86	80.88	78.92
	5–17	75.82	77.12	73.86
MF+MR	5	76.96	79.41	80.88
	10	80.39	79.90	83.33
	17	83.82	84.80	85.78
	5–17	81.05	82.35	79.08
PCA-MF+MR	5	80.39	65.69	82.35
	10	82.35	74.02	83.82
	17	80.88	82.84	86.76
	5–17	79.74	80.72	81.16

**Fig. 11.**
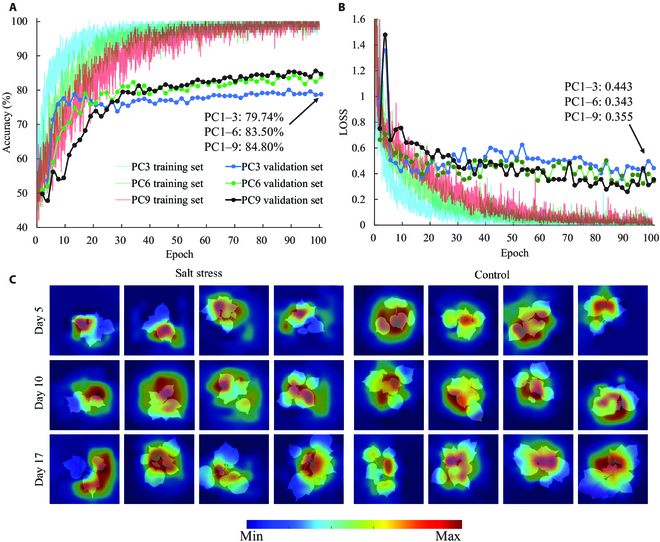
Overall accuracies (A) and losses (B) of training and validation of EfficienNet-B0 with different inputs during model training. Saliency visualization of EfficientNet-B0 for salt stress detection (C).

Besides traditional supervised machine learning approaches, deep learning was also applied to detect salt stress in cotton seedlings due to its ability to extract features automatically with the aim of improving detection performance. In this study, principal component (PC) images from PC1 to PC3, PC1 to PC6, and PC1 to PC9 were used to train EfficientNet-B0, respectively, with the aim of investigating the impact of inputs on model performance. The training performance of EfficientNet-B0 with different inputs is shown in Fig. 11A and B. During the training process, neural networks with fewer inputs generally achieved a lower predictive accuracy. EfficientNet-B0 achieved the best validation accuracy with 3 channels (PC1 to PC3) for the training dataset after approximately 14 epochs. In contrast, using images of PC1 to PC6 and PC1 to PC9 required about 34 and 98 epochs, respectively. For the validation dataset, EfficientNet-B0 obtained the best overall detecting accuracies (whole experimental period) of 79.74%, 83.50%, and 84.80% using PC images from PC1 to PC3, PC1 to PC6, and PC1 to PC9, respectively (Fig. 11A), with a loss of 0.443, 0.343, and 0.355, respectively (Fig. 11B). It can also be found that EfficientNet-B0 (84.80%) with the PC1 to PC9 input outperformed traditional MLs, such as SVM (79.74%), RF (80.72%), and KNN (81.16%) used in this study, which might be due to the fact that it has the ability to automatically learn more abstract and higher-level features about samples. Figure 11C visualizes the important regions within the representative samples for salt stress detection according to the results of gradient-weighted class activation mapping (Grad-CAM) [58]. It revealed that the top leaves of the cotton seedlings’ canopy, particularly those near the young shoots and certain leaf edges, received higher saliency scores for diagnosing salt stress of cotton seedlings.

**Fig. 12.**
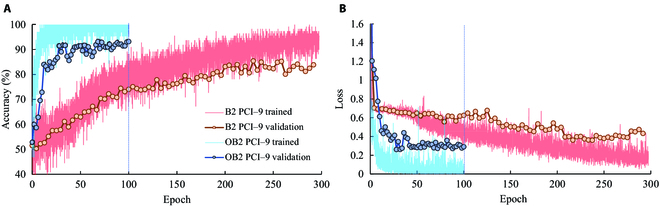
The overall accuracies (A) and losses (B) of the EfficientNet-B2 and EfficientNet-OB2 using 9 principal component images (PC1 to PC9) as input. The weights of PC1 to PC9 in EfficientNet-OB2 were 11, 6, 5, 3, 2, 2, 1, 1 and 1, respectively.

To improve the accuracy, several approaches can be taken into consideration, such as increasing input resolution and expanding the model’s size by increasing depth and width. In this study, the EfficientNet offers a good framework for expanding the model size. Therefore, EfficientNet B2 was further introduced for analysis by applying compound scaling to expand the size of the network for improving detection performance. However, during the EfficientNet B2 training process, it required more training time (230 epochs) to achieve a better detecting performance than that of EfficientNet B0 (84.80% at epoch of 98). This can be attributed to the idea that although different channels have varying levels of importance, each channel was equally treated with the same importance in the first 2D convolutional layer of the input. This led to a decrease in the efficiency of mining the fused images by the convolutional network, which became more problematic with more complex models. Because the amount of information within each channel was different, it was necessary to consider the different importance of each channel during model establishment. To address this issue, an optimized input convolution layer was proposed (Fig. S1). In this method, the image of each sample (including 9 channels from PC1 to PC9) was split into 9 single-channel images as model input, and a proportional number of convolution kernels were assigned to the first convolution of each channel based on the contribution of each channel from the PCA (Table S1). Later, each channel was connected in the direction of the input depth required by the model. The results of salt stress detection of cotton seedlings using general and optimized EfficientNet-B2 models based on 9 principal components’ fused image inputs are shown in Fig. 12A and B. The optimized EfficientNet-B2 (EfficientNet-OB2) presented a significantly faster training speed (14 epochs) than that of general EfficientNet-B2 (118 epochs). Furthermore, the training accuracy of EfficientNet-OB2 achieved 90.35% after 100 epochs, which was 5.31% higher than that of general EfficientNet-B2 (85.01%) after 300 epochs. The optimized method also worked using ResNet-19 and DenseNet-101ResNet-18 as shown in Fig. S2. The training speed of the optimized ResNet-19 and DenseNet-101 increased by more than 50%. It indicated that the optimized input convolutional layer was critical and efficient for the cotton seedlings’ salt stress detection using multichannel images.

The detecting performance of optimized EfficientNet-B2 (EfficientNet-OB2) and general EfficientNet-B2 using the different image channels of each sample as input was compared in Table 3. More image channels of each sample can improve the models’ detecting performance. It can be also observed that optimized EfficientNet-B2 outperformed general EfficientNet-B2. Additionally, the EfficientNet-OB4 was also established and its performance was also discussed. As shown in Table 3, the detecting accuracies of EfficientNet-OB4 were slightly higher than those of EfficientNet-OB2, although it needed more parameters and more training time to establish the model. It demonstrated that EfficientNet-OB2 was more feasible for salt detection of cotton seedlings than EfficientNet-OB4.

**Table 3.** Cotton seedlings salt stress detection performance based on different scales of EfficientNet. Epoch_80%_ is the epoch of EfficientNet reaching the overall accuracy of 80% during the training process.

Model	Input size	Parameters (M)	Epoch_80%_	Epoch time (min)	Mini-batch size	Accuracy (%)	F1 score
EfficientNet-B2 PC1–3	260*260*3*1	6.1	16	4–5	64	81.86	0.812
EfficientNet-B2 PC1–6	260*260*6*1	6.1	54	4–5	64	85.13	0.844
EfficientNet-B2 PC1–9	260*260*9*1	6.1	188	4–5	64	85.46	0.847
EfficientNet-OB2 PC1–3	260*260*1*3	6.1	16	4–5	64	84.80	0.839
EfficientNet-OB2 PC1–6	260*260*1*6	6.1	14	4–5	64	88.40	0.875
EfficientNet-OB2 PC1–9	260*260*1*9	6.1	14	4–5	64	90.36	0.900
EfficientNet-OB4 PC1–3	388*388*1*3	14.6	48	11–12	24	85.62	0.835
EfficientNet-OB4 PC1–6	388*388*1*6	14.6	42	11–12	24	89.22	0.877
EfficientNet-OB4 PC1–9	388*388*1*9	14.6	48	11–12	24	90.89	0.900

The discriminant performance of EfficientNet-OB2 (trained on PC images from PC1 to PC9) for salt-stressed cotton seedlings in different periods is further shown in Table 4. An increasing overall detecting accuracy was found with the extension of salt stress, with values of 84.80%, 91.18%, and 95.10% for 5, 10, and 17 days of salt treatment, respectively, with F1 scores of 0.839, 0.907 and 0.951. Compared to the PCA-KNN method, the EfficientNet-OB2 network improved the detection accuracy by 2.45%, 7.36%, and 8.34% on day 5, day 10, and day 17, respectively, which indicated a priority of EfficientNet-OB2 for the situation of salt detection of cotton seedlings. However, it should also be mentioned that although EfficientNet-OB2 achieved a satisfactory result, it depended on the modification of the architecture of the network by allocating convolutions according to the contribution of each principal component. That is to say, the PCA should be applied to the original image, which consists of several channels, to obtain information on the contribution of each principal component as well as dimensionality reduction. In this study, the original image of each sample is a multi-channel image including multispectral with multicolor fluorescence images (14 channels), which is quite different to those situations where the sample is a 3-channel RGB image. It would make this proposed model not suitable for them. Overall, the results demonstrated that combining multicolor fluorescence–multispectral reflectance imaging with EfficientNet-OB2 deep learning presented an alternative to screening salt stress of cotton seedlings in the field.

**Table 4.** Discriminant performance of EfficientNet-OB2 trained on images from PC1 to PC9 for salt stress detection of cotton seedlings in different periods

Day	Control (%)	Salt stress (%)	Accuracy (%)	Precision (%)	F1 score
5	88.46	81.00	84.80	87.10	0.839
10	94.23	88.00	91.18	93.62	0.907
17	93.27	97.00	95.10	93.27	0.951
5–17	91.99	88.67	90.36	91.41	0.900

## Conclusion

In this study, a prototyping platform was developed to synchronously collect time-series multicolor fluorescence and multispectral reflectance images of cotton seedlings after 5, 10, and 17 days of salt stress. The Relief algorithm and PCA were used to reduce data dimension with the first 9 principal component images (PC1 to PC9) of each sample accounting for 95.2% of the original variations, which were used for model establishment. An optimized EfficientNet-B2 (EfficientNet-OB2) that assigned the proportional number of convolution kernels to the first convolution according to the contribution of each channel from the PCA results was proposed for salt stress detection of cotton seedlings. It was found that EfficientNet-OB2 training on the first 9 principal component images (PC1 to PC9) achieved the best discriminant accuracies of 84.80%, 91.18%, and 95.10% for 5, 10, and 17 days of salt treatment, respectively. It presented a better result than traditional machine learning methods such as PCA-KNN and EfficientNet-B0. Additionally, it also achieved superior performance with a satisfying overall accuracy of 90.36% with fewer model parameters and less training time in comparison with EfficientNet-B2 and EfficientNet-OB4. Possibly, this method can be applied to screen the salt stress of cotton seedlings in mobile platforms. Further research will pay attention to modifying the structure of the platform to meet the field situation as well as transfer and deploy the model for cotton seedlings’ salt stress detection under field conditions.

## Data Availability

The code and training script of EfficientNet-OB2 has been hosted to GitHub and is available at https://github.com/foddcus/EfficientNetOB.
