# Supplementary material for: Noninvasive Detection of Salt Stress in Cotton Seedlings by Combining Multicolor Fluorescence–Multispectral Reflectance Imaging with EfficientNet-OB2
Source: Plant Phenomics. 2023 Dec 8;5:0125. doi: 10.34133/plantphenomics.0125 (PMC10709074; doi:10.34133/plantphenomics.0125)

**Noninvasive detection of salt stress in cotton seedlings by combining multicolor fluorescence-multispectral reflectance imaging with deep learning**

Jiayi Li ^1,2^, Haiyan Zeng^1,2^, Chenxin Huang^2^, Libin Wu^1,2^, Jie Ma^2^, Beibei Zhou^3^, Dapeng Ye ^1,2^, Haiyong Weng ^1,2*^

^a^ College of Mechanical and Electrical Engineering, Fujian Agriculture and Forestry University, Fuzhou 350002, China

^b^ Fujian Key Laboratory of Agricultural Information Sensoring Technology, College of Mechanical and Electrical Engineering, Fujian Agriculture and Forestry University, Fuzhou, Fujian 350002, China

^c^ State Key Laboratory of Eco-hydraulics in Northwest Arid Region of China, Xi’an University of Technology, Xi’an 710048, Shaanxi, China

*Corresponding author: [ydp@fafu.edu.cn (Dapeng](mailto:ydp@fafu.edu.cn%20(Dapeng) Ye); [hyweng@fafu.edu.cn](mailto:hyweng@fafu.edu.cn) (Haiyong Weng).

**Table S1**. Contribution of each parameter based on PCA

|  | PC1 | PC2 | PC3 | PC4 | PC5 | PC6 | PC7 | PC8 | PC9 | PC10 |
| --- | --- | --- | --- | --- | --- | --- | --- | --- | --- | --- |
| F520 | 0.144537 | 0.105445 | -0.05014 | -0.12854 | 0.36323 | 0.330107 | -0.34051 | -0.23179 | 0.52302 | 0.078441 |
| F690 | 0.175592 | 0.151109 | 0.174006 | -0.31967 | 0.045895 | 0.23334 | 0.138877 | 0.008176 | 0.161437 | 0.009838 |
| F740 | 0.197257 | 0.150315 | 0.108687 | -0.30104 | -0.08647 | 0.328009 | -0.0947 | -0.06031 | 0.031196 | 0.080113 |
| R460 | 0.149346 | -0.21455 | -0.25951 | 0.113213 | -0.01849 | 0.238459 | 0.110225 | 0.186274 | -0.02593 | -0.1073 |
| R520 | 0.308836 | -0.06868 | -0.06068 | 0.120636 | 0.076883 | 0.03436 | 0.023405 | 0.084191 | -0.05001 | -0.03001 |
| R590 | 0.310581 | -0.05317 | -0.0856 | 0.012951 | 0.114617 | -0.07773 | -0.0293 | 0.002362 | -0.12715 | -0.12608 |
| R660 | 0.075996 | 0.300278 | -0.08691 | 0.101561 | 0.310544 | 0.137312 | -0.013 | 0.23352 | -0.20185 | -0.06696 |
| R710 | 0.231905 | -0.05588 | -0.29269 | -0.08926 | 0.096485 | -0.03996 | 0.103249 | -0.00809 | -0.06262 | 0.091189 |
| R730 | 0.29022 | -0.04213 | -0.08567 | 0.179233 | 0.117821 | 0.108323 | 0.141734 | -0.08232 | -0.12985 | -0.01685 |
| R760 | 0.27853 | 0.090151 | -0.0919 | 0.139735 | -0.18098 | 0.021524 | 0.026839 | -0.01432 | 0.129539 | -0.24476 |
| R780 | 0.152703 | 0.276034 | -0.16458 | 0.116846 | -0.07291 | -0.14801 | 0.023466 | 0.068378 | 0.012616 | 0.119811 |
| R810 | 0.085575 | 0.28485 | -0.1603 | 0.137313 | -0.05908 | -0.19614 | 0.01847 | 0.1344 | -0.01099 | 0.326751 |
| R850 | 0.188348 | 0.184823 | -0.21046 | 0.095192 | -0.20541 | -0.1149 | 0.139953 | -0.04197 | 0.184921 | 0.186669 |
| R910 | 0.210389 | 0.189814 | -0.12111 | 0.191517 | -0.21326 | 0.024493 | -0.02802 | -0.01127 | 0.1432 | -0.42249 |
| F520/F690 | -0.13562 | -0.12186 | -0.21367 | 0.31293 | 0.1937 | -0.06714 | -0.32968 | -0.14569 | 0.12 | 0.044945 |
| F520/F740 | -0.15503 | -0.12284 | -0.14328 | 0.28621 | 0.350729 | -0.19155 | -0.06615 | -0.07616 | 0.28307 | -0.05466 |
| F690/F740 | -0.02854 | 0.010368 | 0.177744 | -0.09149 | 0.34871 | -0.25693 | 0.625914 | 0.174848 | 0.393004 | -0.22296 |
| GR | 0.252349 | -0.1037 | 0.170211 | 0.007915 | -0.07161 | -0.25472 | -0.04825 | -0.17716 | 0.116077 | 0.14603 |
| PSRI | -0.16633 | 0.322284 | -0.05906 | 0.010338 | 0.034507 | -0.05263 | -0.01289 | 0.067766 | -0.02964 | 0.119969 |
| R460/R520 | -0.20605 | -0.18768 | -0.22643 | -0.03412 | -0.10745 | 0.21738 | 0.101976 | 0.094859 | 0.026532 | -0.0741 |
| R460/R590 | -0.06992 | -0.04773 | 0.109 | 0.39012 | -0.15113 | 0.402164 | 0.197571 | 0.257469 | 0.278608 | 0.372391 |
| R460/R730 | -0.1623 | -0.2296 | -0.21397 | -0.09507 | -0.14738 | 0.140118 | -0.03538 | 0.305803 | 0.10701 | -0.10095 |
| R520/R660 | 0.16224 | -0.30537 | 0.034884 | -0.02868 | -0.19185 | -0.08513 | 0.020918 | -0.14986 | 0.146425 | 0.03222 |
| R520/R710 | 0.050105 | -0.00651 | 0.380104 | 0.278782 | -0.04664 | 0.045674 | -0.0411 | 0.149544 | 0.057071 | 0.156006 |
| R520/R730 | 0.159169 | -0.11361 | 0.06649 | -0.17315 | -0.11619 | -0.26612 | -0.42262 | 0.622657 | 0.257465 | -0.05358 |
| R520/R850 | 0.203256 | -0.23426 | 0.084126 | 0.038615 | 0.262041 | 0.113105 | -0.06801 | 0.153796 | -0.21908 | -0.11751 |
| R520/R910 | 0.184496 | -0.24939 | 0.012162 | -0.05074 | 0.28014 | -0.0086 | 0.074665 | 0.128941 | -0.18266 | 0.437836 |
| R590/R710 | 0.095579 | 0.016086 | 0.397624 | 0.14135 | 0.021783 | -0.1403 | -0.16131 | 0.042127 | -0.08217 | -0.05367 |
| R660/R730 | -0.12866 | 0.314707 | -0.01798 | -0.03412 | 0.212603 | 0.059594 | -0.11799 | 0.271281 | -0.09588 | -0.10122 |
| R710/R730 | -0.00202 | -0.04122 | -0.31835 | -0.36116 | 0.019425 | -0.1958 | 0.021232 | 0.063424 | 0.073387 | 0.244735 |

**Table S2.** Parameters of machine learning models after optimizing.

|  | SVM | | | RF | | KNN | | |
| --- | --- | --- | --- | --- | --- | --- | --- | --- |
|  | Kernel | Gamma | C | Number of estimators | Max depth | Distance metric | Distance weight | Number of neighbors |
| MF | Quadratic | 1 | 75.5356 | 455 | 10 | Chebyshev | Equidistant | 17 |
| MR | Linear | 1 | 0.1119 | 19 | 4 | City-block | Inverse distance | 37 |
| MF+MR | Linear | 1 | 0.39173 | 499 | 447 | Correlation | Inverse distance | 28 |
| PCA-MF+MR | Linear | 1 | 0.6381 | 458 | 30 | Hamming | Inverse distance | 15 |

Note: *C* is the regularization in SVM


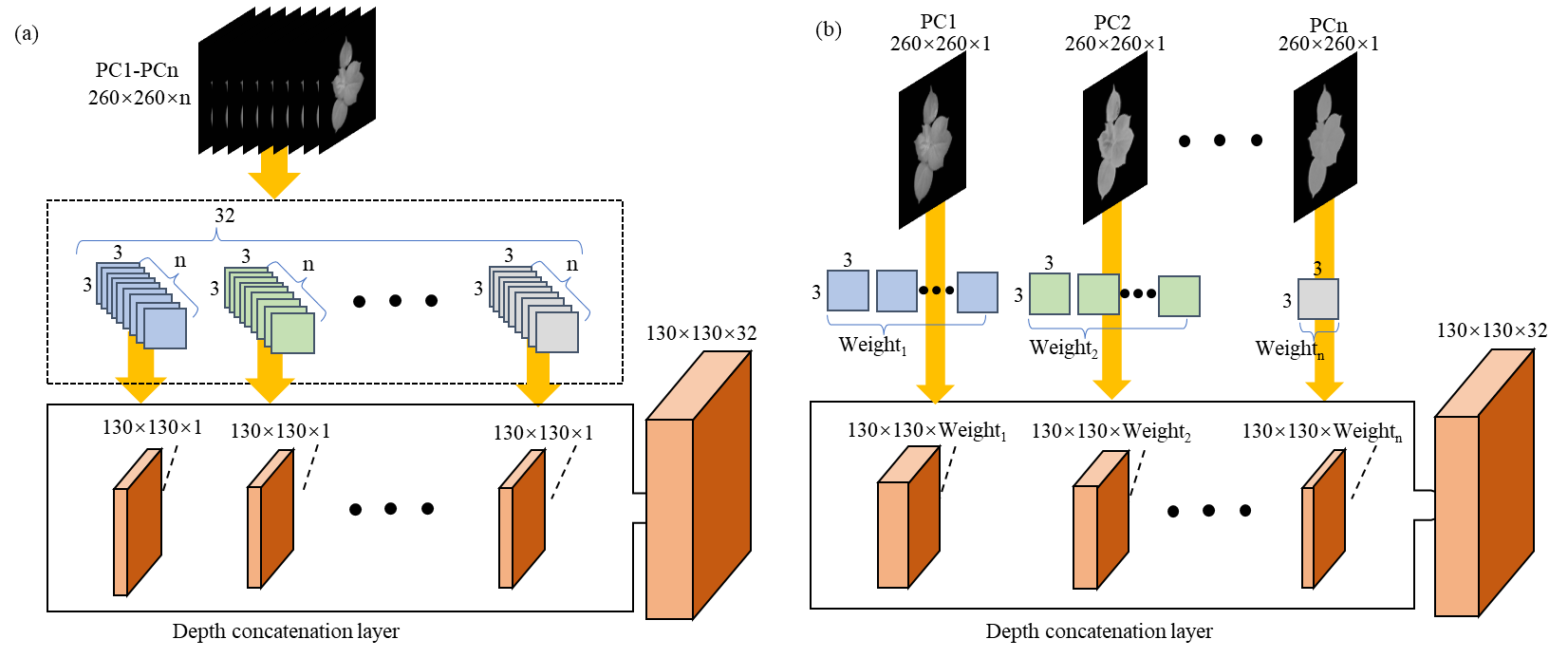


**Figure S1**. The input convolutional layer of general EfficientNet-B2 input convolutional layer (a) and optimized EfficientNet-B2 (OB2) (b). The stride of a convolutional layer is set as 2. *Weight_n_* represents the number of filters in convolution assigned based on the interpretability of the principal components *n*. The weight of PC1 to PC9 is 11, 6, 5, 3, 2, 2, 1, 1 and 1, respectively.

**Figure S2.** The performance of optimized ResNet-19 (a) and optimized DenseNet-

121(b)using our proposed model.


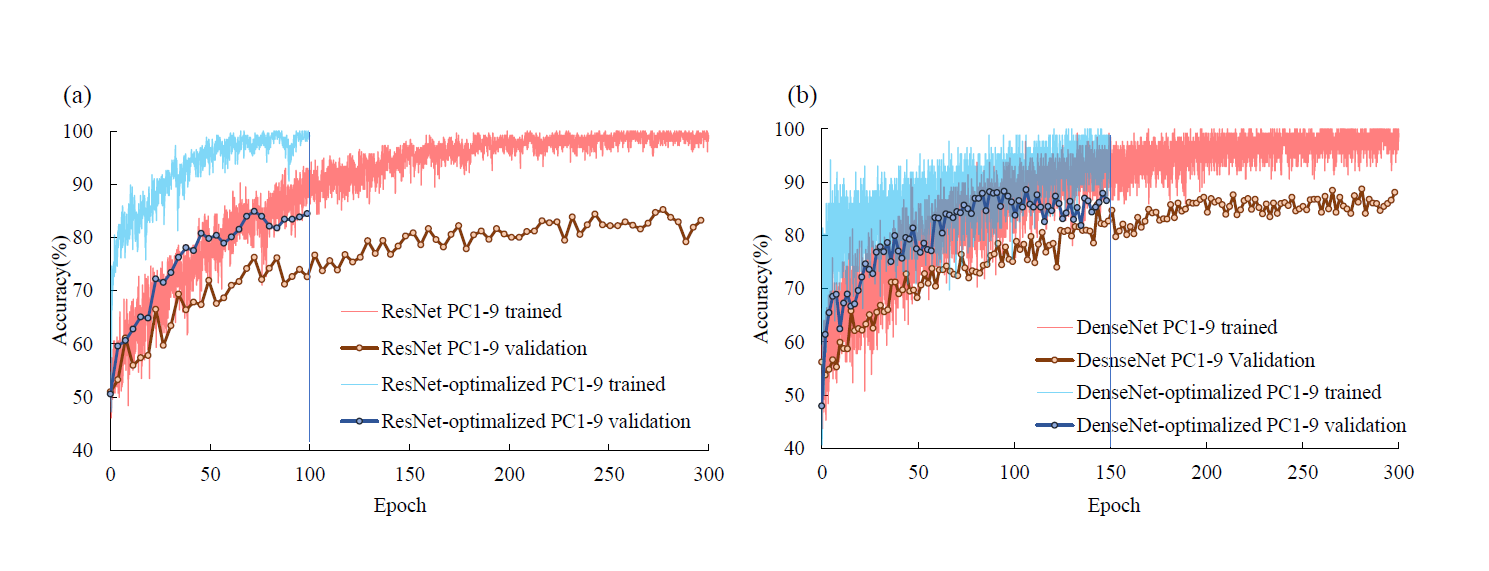

Supplement: Supplementary 1 — Figs. S1 and S2 Tables S1 and S2 [file plantphenomics.0125.f1.zip › Supplementary material final.docx]
