# Supplementary figures and images for: Noninvasive Detection of Salt Stress in Cotton Seedlings by Combining Multicolor Fluorescence–Multispectral Reflectance Imaging with EfficientNet-OB2
Source: Plant Phenomics. 2023 Dec 8;5:0125. doi: 10.34133/plantphenomics.0125 (PMC10709074; doi:10.34133/plantphenomics.0125)

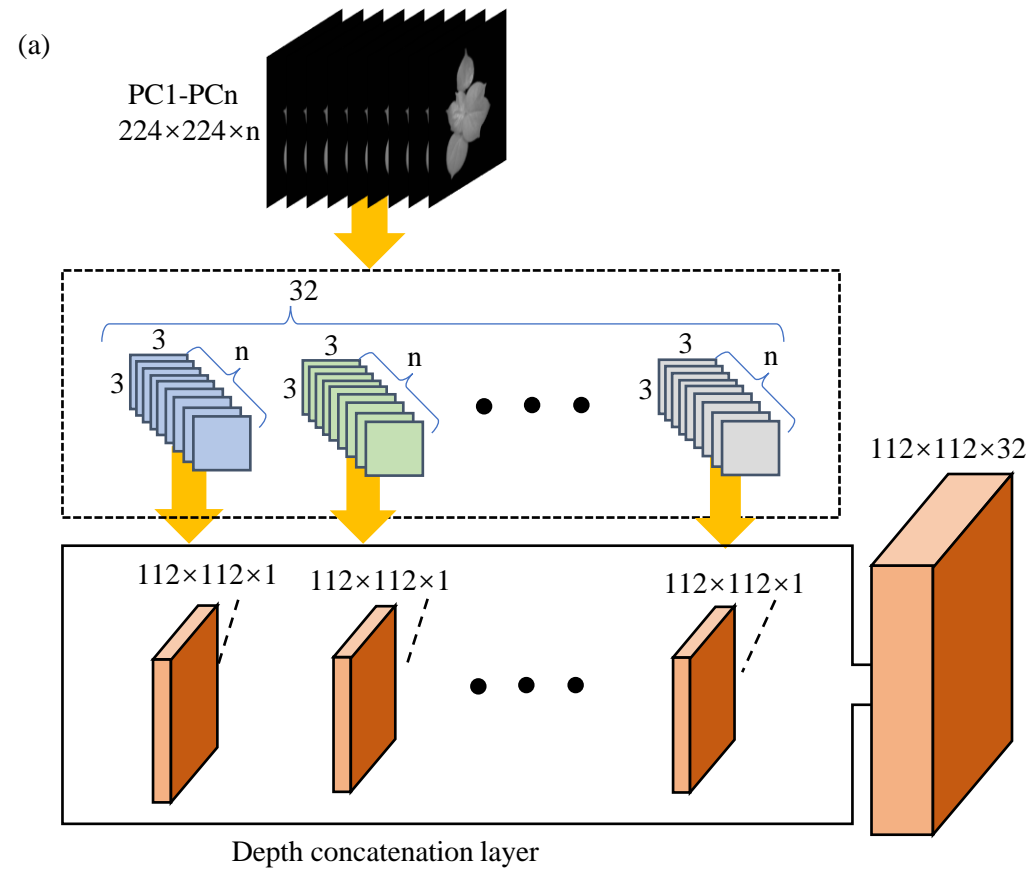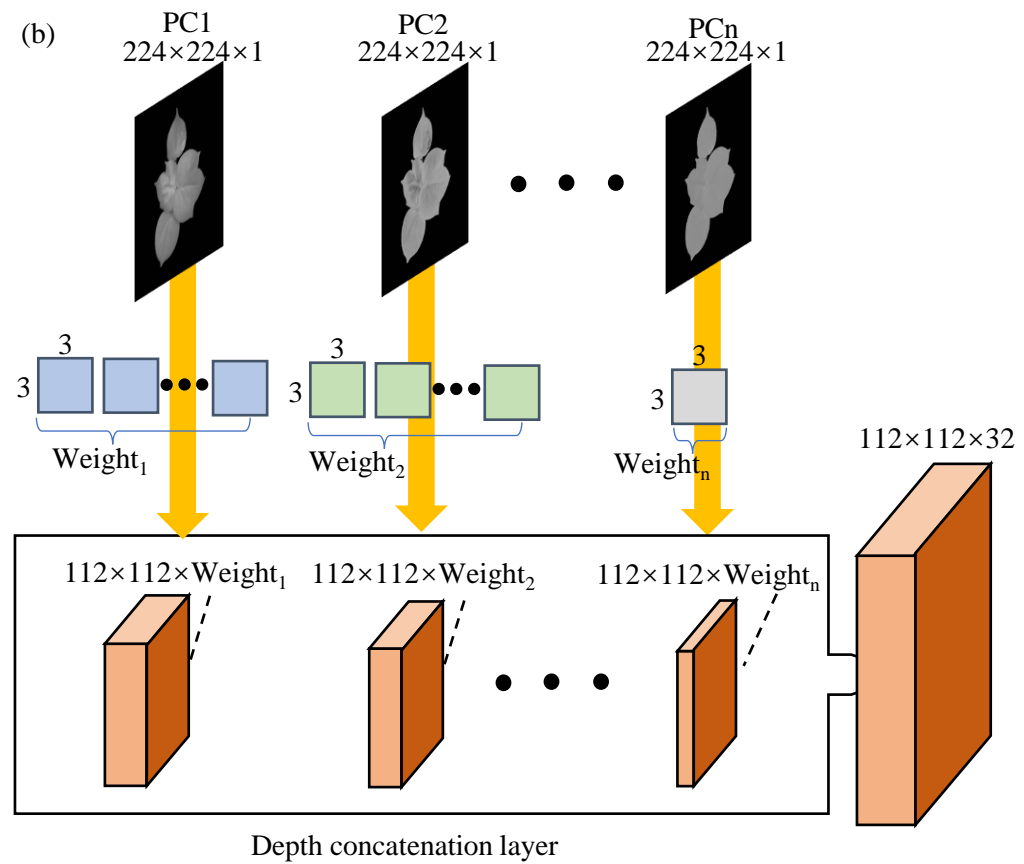

Supplement: Supplementary 1 — Figs. S1 and S2 Tables S1 and S2 [file plantphenomics.0125.f1.zip › Figure S1.pdf]

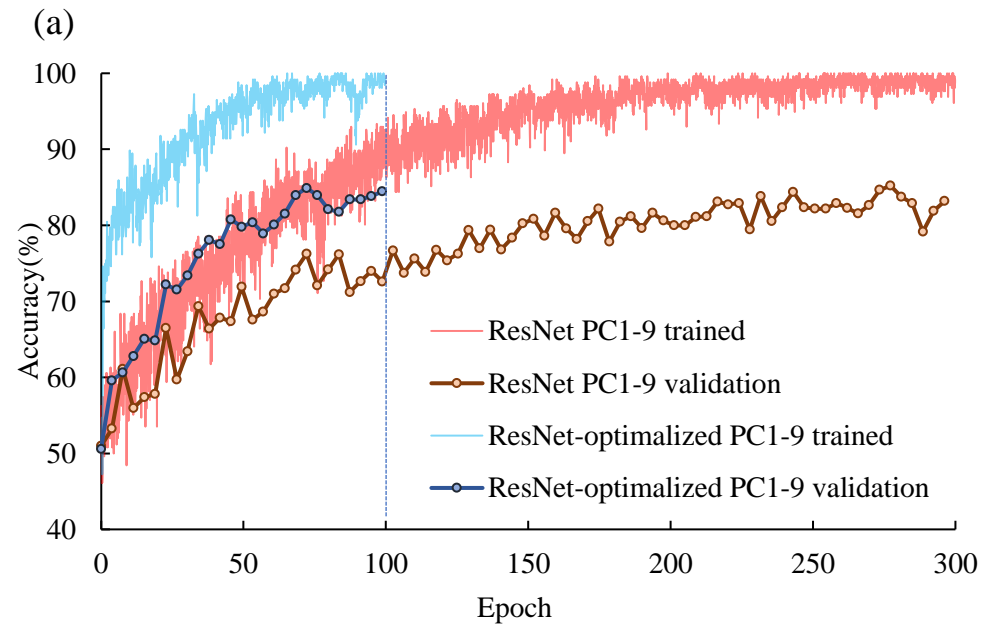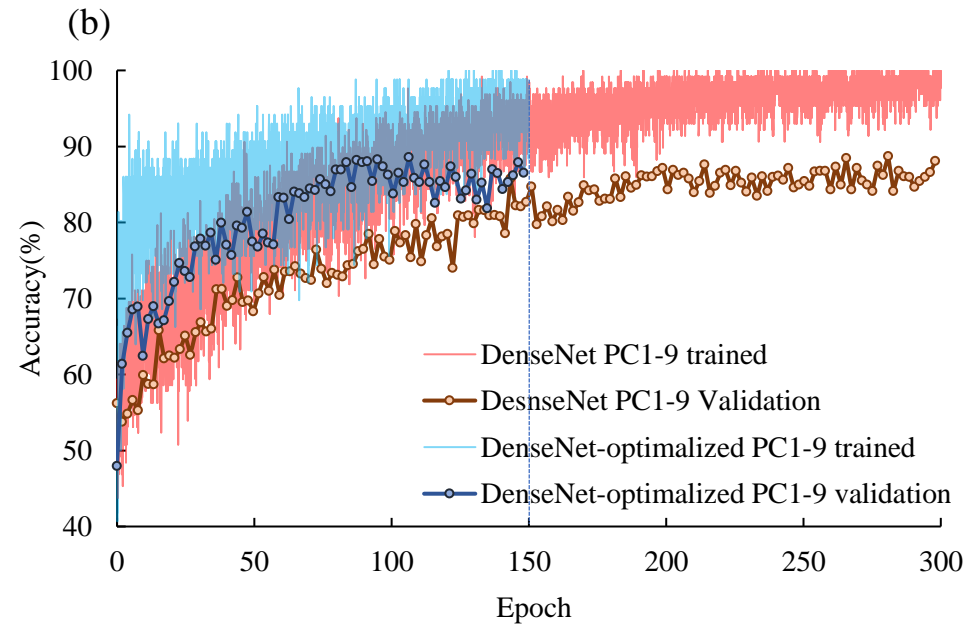

Supplement: Supplementary 1 — Figs. S1 and S2 Tables S1 and S2 [file plantphenomics.0125.f1.zip › Figure S2.pdf]
